# Complete Genome Sequence of Pseudomonas aeruginosa K34-7, a Carbapenem-Resistant Isolate of the High-Risk Sequence Type 233

**DOI:** 10.1128/MRA.00886-18

**Published:** 2018-08-02

**Authors:** George Taiaroa, Ørjan Samuelsen, Tom Kristensen, Ole Andreas Løchen Økstad, Adam Heikal

**Affiliations:** a Department of Microbiology and Immunology, School of Biomedical Sciences, University of Otago, Dunedin, New Zealand; b Department of Biochemistry, School of Biomedical Sciences, University of Otago, Dunedin, New Zealand; c Norwegian National Advisory Unit on Detection of Antimicrobial Resistance, Department of Microbiology and Infection Control, University Hospital of North Norway, Tromsø, Norway; d Department of Pharmacy, Faculty of Health Sciences, UiT—The Arctic University of Norway, Tromsø, Norway; e Department of Biosciences, University of Oslo, Oslo, Norway; f Centre for Integrative Microbial Evolution (CIME), Faculty of Mathematics and Natural Sciences, University of Oslo, Oslo, Norway; g Laboratory for Microbial Dynamics (LaMDa), Section for Pharmaceutical Biosciences, School of Pharmacy, University of Oslo, Oslo, Norway; University of Arizona

## Abstract

Carbapenem-resistant Pseudomonas aeruginosa is defined as a “critical” priority pathogen for the development of new antibiotics. Here we report the complete genome sequence of an extensively drug-resistant, Verona integron-encoded metallo-β-lactamase-expressing isolate belonging to the high-risk sequence type 233.

## ANNOUNCEMENT

Carbapenem-resistant Pseudomonas aeruginosa is a critical threat to public health (1). P. aeruginosa K34-7 belongs to sequence type 233 (ST233) and is an extensively drug-resistant (XDR), carbapenem-resistant clinical isolate expressing the Verona integron-encoded metallo-β-lactamase (VIM-2) (2). ST233 has been identified as a high-risk clone in both Mexico (3) and the United States (4). K34-7 was the first metallo-β-lactamase-producing P. aeruginosa isolate identified in Norway, and PCR analysis previously confirmed that the *bla*_VIM-2_ gene was contained within an unusual class 1 integron (GenBank accession number FM165436) (2). As only one other P. aeruginosa ST233 complete genome has been published (5), this high-quality P. aeruginosa K34-7 genome will provide a valuable additional genomic resource for investigation of this high-risk ST.

Genomic DNA was prepared from a culture grown from a single colony using the Mo Bio DNeasy UltraClean microbial kit (Qiagen, USA) and sequenced on a PacBio RS II platform. A standard library of 20-kb fragments was prepared using the BluePippin preparative electrophoresis system (Sage Science, USA) with a 9-kb cutoff and sequenced on a single-molecule real-time (SMRT) cell using P6-C4 chemistry with 360-min movie-time chemistry. Additional whole-genome sequencing (WGS) was performed using an Illumina HiSeq sequencer. Genome assembly involved a *de novo* approach, using default HGAP 4 settings for the assembly of 96,269 PacBio reads (average length, 10,760 bp), before manual curation and validation. Iterative read mapping of Illumina sequences using custom settings in Geneious 10.1.3 (6) was used to identify assembly errors, primarily single-base insertions and deletions, and for variant correction (0.7 minimum variant frequency, 5× minimum coverage). Custom settings included allowed gaps (15% maximum/read and 15-bp maximum size); word and index word lengths of 18 and 13, respectively; and 20% maximum mismatch/read and maximum ambiguity of 4.

The K34-7 genome consists of a 7,038,012-bp chromosome and one plasmid, pK34-7-1 (4,440 bp). A P. aeruginosa genomic island 5 (PAGI-5)-like hybrid (7) pathogenicity island (84,893 bp) and bacteriophage (38,832 bp) were found on the chromosome (positions 4154015 to 4238618 and 6469185 to 6508016, respectively). The XDR K34-7 phenotype is predominantly due to genes associated with three chromosomally located class 1 integrons, including genes imparting resistance to aminoglycosides [*aac(3)*, *aac(3)-I*, *aac(6´)-Il*, *aadA2*, and *aph(3´)-IIb*], β-lactams (*bla*_OXA-4_, *bla*_OXA-486_, *bla*_PDC-3_, and *bla*_VIM-2_), chloramphenicol (*catB*, *cmlA6*, and *floR*), trimethoprim (*dfrB5*), fosfomycin (*fosA*), sulfonamide (*sul1*), and tetracycline (*tetG*), as annotated by the PGAP pipeline (8). Additionally, *tetK*, encoding the tetracycline efflux pump TetK, is found on the small pK34-7-1 plasmid.

A region (1936000 to 2043700) of 12 direct tandem repeats (7,122 bp, 11.5× mean Illumina coverage) encodes a zonular occludens toxin, previously characterized in Vibrio cholerae (9, 10). Similar regions appear in other P. aeruginosa genomes but not in the ST233 P. aeruginosa PA83 genome (5). Additionally, P. aeruginosa K34-7 contains a type I-F CRISPR-Cas system (1656563 to 1664702), previously described in P. aeruginosa strain UCBPP-PA14 (11).

Complete high-quality bacterial genomes facilitate further research into mechanisms of resistance and their dissemination and aid in the development of new therapies for XDR infections.

### Data availability.

This complete genome project has been deposited at GenBank under the accession numbers CP029707 and CP029708.
